# Simple Incisionless Temporary Stabilization: An Adjunct to Lower Blepharoplasty

**DOI:** 10.1093/asjof/ojae014

**Published:** 2024-03-13

**Authors:** Sara N Reggie, Tiffany C Ho, Adam G Buchanan, John B Holds

## Abstract

**Background:**

Lateral canthal tightening is indicated for patients undergoing lower eyelid blepharoplasty who have preexisting lower eyelid laxity or ectropion. A canthoplasty or canthopexy is indicated at the time of lower blepharoplasty to avoid postoperative complications, such as eyelid retraction or ectropion. Various surgical techniques are described to accomplish this goal, including canthopexy procedures, which usually access the lateral canthal tendon through an upper eyelid blepharoplasty or lateral canthal incision.

**Objectives:**

To describe an incisionless technique adjunctive to lower blepharoplasty, which stabilizes the lower eyelid in the week following blepharoplasty.

**Methods:**

This operative technique description and retrospective case series includes 15 patients who underwent a simple incisionless temporary stabilization (SITS) during lower eyelid blepharoplasty. The procedures were performed at the same outpatient office-based surgery center and were performed by the author surgeons. Patients were followed from 3 to 6 months postoperatively.

**Results:**

The SITS procedure during lower eyelid blepharoplasty successfully maintained a desirable functional and aesthetic eyelid position with minimal complications. One patient reported tearing postoperatively which was determined to be unrelated to the SITS and resolved by the 1-month follow-up visit. No patient had any other complications during the follow-up period.

**Conclusions:**

The SITS procedure was successfully utilized in patients with mild-to-moderate lower eyelid laxity and/or a negative vector to prevent postoperative ectropion and eyelid retraction. It is a more favorable alternative to temporary tarsorrhaphy, as it does not obstruct vision during healing and better secures the eyelid. It should not be used in patients with significant lower eyelid laxity that would place the patient at significant risk of ectropion and lower eyelid retraction related to swelling and inappropriate eyelid position during the early postoperative course.

**Level of Evidence: 4:**

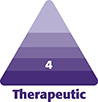

Lower eyelid ectropion, laxity, and retraction are common causes of tearing and/or ocular surface irritation and can have both functional and cosmetic consequences, especially after lower eyelid blepharoplasty. Failure to recognize these issues on preoperative assessment may result in eyelid retraction or worsening of ectropion postoperatively. Addressing lower eyelid ectropion and laxity may be accomplished through a lateral canthoplasty or canthopexy, depending on the severity of lateral canthal tendon (LCT) laxity and/or disinsertion. Horizontal tightening of the lower lid remains the fundamental solution to these problems and assists in proper eyelid closure. Various surgical techniques have been described to accomplish this goal, including canthopexy procedures which usually access the LCT through an upper eyelid blepharoplasty or lateral canthal incision.^1-6^ More invasive canthoplasty procedures are generally indicated to provide robust lower eyelid support in more profound cases of retraction and laxity. Many patients with mild to moderate laxity still benefit from a canthopexy procedure that provides temporary support to the eyelid in the early postoperative period in which swelling may evert the eyelid margin or push the eyelid margin inferiorly. Temporary tarsorrhaphy is another alternative, although this will significantly narrow the horizontal and vertical eyelid fissure and does not tighten horizontally, even temporarily.

In this study, we describe a simple incisionless temporary stabilization (SITS), performed without upper blepharoplasty or canthal incisions, which helps secure an optimal lower eyelid positioning postlower blepharoplasty.

## METHODS

A retrospective chart review was performed on all patients who had a SITS during a lower eyelid blepharoplasty at our practice from October 2019 to January 2023. All information was gathered in a Health Insurance Portability and Accountability Act-compliant fashion, and the report adhered to the ethical principles as outlined in the Declaration of Helsinki as amended in 2013. It was determined by the Institutional Review Board (IRB) that our study was eligible for an IRB waiver as it was retrospective and complied with proper consideration for the rights and welfare of human patients.

Our technique was utilized in patients undergoing a transconjunctival lower eyelid blepharoplasty with fat repositioning and who had preexisting mild-to-moderate lower eyelid laxity and/or a negative facial vector. All surgeries occurred in the same outpatient office-based surgery center by one of the authors.

**Figure 1.**
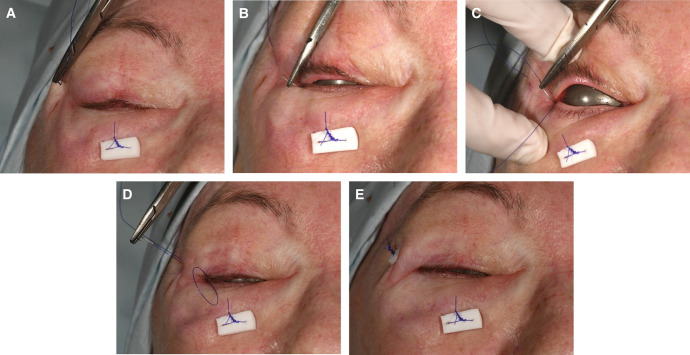
(A) A 61-year-old female patient directly following lower eyelid transconjunctival blepharoplasty with fat transposition. Fat is internally transposed over the orbital rim, with suture exiting the midface externally and tied over a foam bolster as pictured (separate from the simple incisionless temporary stabilization [SITS] procedure). For the SITS procedure, a 4-0 polydioxanone on an RB-1 needle is passed through the temporal upper eyelid skin, capturing a small amount of orbital rim periosteum. (B) The needle exits the canthal angle. (C) The needle is reversed and passed back through the canthal angle and upper eyelid. (D) The needle then exits back out through the temporal lid skin. (E) The knot is secured over a bolster.

No skin incisions are necessary. A 4-0 polydioxanone suture with an RB-1 needle (Ethicon, Raritan, NJ) is first passed through the skin and orbicularis muscle of the temporal upper eyelid crease, capturing a small amount of superior orbital rim periosteum, then passes inferiorly underneath the skin and orbicularis muscle (Figure 1A). This pass must be superficial as to avoid capturing levator aponeurosis. The needle exits through the lateral canthal angle at the mucocutaneous junction of the lower eyelid (Figure 1B).

The needle is then entered back into the lateral canthal angle at the same location as the original pass (Figure 1C). It then travels upward, underneath skin and orbicularis muscle of the upper eyelid, capturing a small amount of superior rim periosteum, then out through the skin 3 mm from the entrance point (Figure 1D). The suture is tied off over a bolster and trimmed (Figure 1E). Care must be taken to titrate the tightening suture as overtightening can cause dimpling and exaggerated upward slanting of the lower eyelid (which is only temporary). The SITS suture and bolster are left in place for 5 to 7 days, depending on appointment time/availability, and are removed easily at a postoperative visit. Please see Video demonstration for reference.

## RESULTS

**Figure 2.**
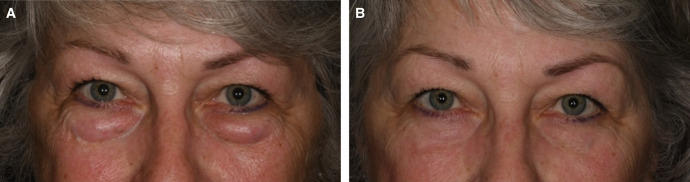
(A) The preoperative photograph of a 65-year-old female with mild-to-moderate lower eyelid laxity and (B) postoperative photograph of the same patient 6 months after lower eyelid transconjunctival blepharoplasty with fat transposition and simple incisionless temporary stabilization.

**Figure 3.**
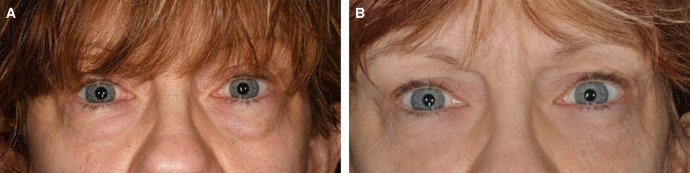
(A) The preoperative photograph of a 64-year-old female with mild-to-moderate lower eyelid laxity and (B) postoperative photograph of the same patient 3 months after lower eyelid transconjunctival blepharoplasty with fat transposition and simple incisionless temporary stabilization.

Fifteen patients (13 females and 2 males) underwent the SITS procedure in conjunction with a lower eyelid transconjunctival blepharoplasty with fat repositioning. The mean age was 63 years, with a range of 43 to 82 years. Patients were evaluated in the 3- to 6-month postoperative period, with a mean period of 4.2 months. Preoperative and postoperative evaluations were performed by the primary surgeon and included an anterior segment ophthalmological examination and a comprehensive eyelid examination including lateral canthal tilt, lower eyelid position in relation to the corneoscleral limbus, snap-back and eyelid distraction tests, and facial vector. Preoperative eyelid laxity was determined to be mild (1-2 mm) or moderate (3-5 mm) by using the lid distraction test.^5^ Patients with severe laxity (6 mm or greater) on lid distraction testing were excluded as they required a lateral canthoplasty procedure. Six patients (40%) had positive lateral canthal tilt preoperatively, and 9 (60%) had neutral lateral canthal tilt preoperatively. Three (20%) patients were noted to also have a negative facial vector preoperatively. Patients with mild to moderate eyelid laxity underwent a SITS procedure during transconjunctival blepharoplasty with fat repositioning (Figures 2, 3). All 15 patients were noted to have optimal functional and aesthetic lower eyelid positioning postoperatively, which the authors defined as the lower eyelid margin resting at the inferior corneoscleral limbus with its lowest point slightly temporal to the pupil center, as well as the absence of scleral show, frank ectropion, and eyelid retraction.^5^ Canthal tilt measurements remained stable in all patients. Only 1 patient reported tearing postoperatively. This was determined to be caused by the patient's preexisting dry eye syndrome and unrelated to the SITS suture, and it resolved within 1 week with aggressive artificial tear usage. No patients reported ocular irritation or had examination findings consistent with ocular exposure. No other complications including canthal dystopia, webbing, or scarring occurred.

## DISCUSSION

Involutional ectropion is customarily treated by tightening the lower eyelid horizontally and anchoring the LCT to the inner periosteum of the lateral orbital rim. This may be done through canthoplasty, which requires a canthotomy and cantholysis prior to lower tarso-ligamentous resuspension.^7-11^ In contrast, a canthopexy is a less invasive means of canthal resuspension and tightens the LCT without incising the canthal angle; however, there is usually a permanent suture or technique that encourages the formation of a firm supporting adhesion in the lateral canthal region.

Firm attachment of the LCT to the inner lateral orbital rim is crucial for appropriate lower eyelid position and thus optimal ocular health. Surgeons must recognize ectropion and lower eyelid laxity on preoperative assessment to avoid complications. This may be accomplished primarily through the lid distraction and snap-back tests, as well as other clinical features, such as medial and inferior movement of the lateral commissure with eyelid closure, a blunted lateral canthal angle and resultant horizontally reduced visible temporal scleral wedge, incomplete apposition of the eyelid margins without anterior lamellar shortage, and eyelid imbrication.^12-14^ If these issues are not recognized preoperatively and addressed at the time of lower eyelid blepharoplasty, ectropion, eyelid retraction, “fishmouthing syndrome,” tearing, and ocular exposure and irritation may ensue.^12,13^

There are many surgical variations of stabilizing the LCT used during reconstructive and aesthetic eyelid surgery, including the classic lateral tarsal strip (LTS) procedure, the inferior retinacular canthoplasty, canthal sparing lateral canthal resuspension, the transblepharoplasty canthopexy, and the temporary Frost suture or tarsorrhaphy.^1-4,6,7,10,15^

The LTS procedure was described by Anderson and Gordy and is utilized to treat senile, cicatricial, and paralytic ectropion caused by disinsertion and/or stretching the LCT.^7^ It is also a useful approach in treating postblepharoplasty lower eyelid malposition. Since its description in 1979, this procedure has effectively been shown to stabilize the lower eyelid while giving the lateral canthal angle a natural appearance. Although it is very useful in cases of severe eyelid laxity and frank ectropion, it has a longer healing time with pain and swelling and may distort the canthus in some surgeons' hands.^7,10^

Canthal suspension or minimally invasive canthopexy alone may suffice when the lower lid has minimal laxity, and many canthal resuspension techniques have been described that use an upper eyelid blepharoplasty incision as an entry point.^1-4,10,14^ These approaches are especially attractive options for aesthetic patients, given the low risk of long-term healing issues or issues with patient satisfaction. In 1996, Jelks et al described an inferior retinacular lateral canthoplasty. This procedure consists of tightening the lateral canthus through the lateral aspect of an upper eyelid blepharoplasty incision.^10^

Additionally, a temporary Frost suture involves passing a suture through the lower eyelid margin and taping it to the forehead for 3 to 5 days. This provides upward traction on the lower eyelid as well as more completely closes the eyelid aperture aiding in treating chemosis; however, it is not as secure as methods discussed above, does not provide horizontal tightening, and may also obstruct the patient's vision while in place.^15^

Our SITS technique is simple, has the advantage of requiring no upper blepharoplasty incision, does not cause visual obstruction, and securely stabilizes the lower eyelid during the healing process postblepharoplasty by providing an upward and horizontal tension vector in mild-to-moderately lax eyelids that may retract downward during the initial healing process. This technique also successfully prevents imbrication of the upper and lower eyelids, as the apex of the canthal angle is being suspended and traction is applied to both the upper and lower cruxes of the tendon. Limitations of our study include a small sample size, different surgeons, and the dependency on the accuracy of medical records for gathering patient information. Additionally, our technique's purpose is not to prevent chemosis, as it does not completely close the eyelid aperture. Chemosis should be treated with other modalities, such as aggressive lubrication, topical steroids, conjunctival cautery, and/or Frost suture.

It can be argued that a canthopexy involves the use of a suture or technique that encourages the formation of a firm supporting adhesion in the lateral canthal region; therefore, we refer to our technique as a “stabilization.” We believe that SITS serves as a valuable adjunct to lower eyelid blepharoplasty in the immediate postoperative period when edema and fibrin reaction are highest, increasing the risk for long-term or permanent eyelid retraction. Given the simple and low-risk nature of this procedure, as well as the authors' consistent and reproducible results, the authors recommend a low threshold for performing SITS during lower blepharoplasty in patients with preexisting mild-to-moderate lid laxity and/or negative facial vector.

## CONCLUSION

SITS is optimally utilized in patients with mild-to-moderate lower eyelid laxity and/or a negative vector in which upward tension on the lower eyelid during the initial healing window will aid in preventing downward retraction. It should not be used in patients with marked lower eyelid laxity who would benefit more from a horizontal tightening canthoplasty procedure, such as a LTS procedure.
